# Pace, pause & silence: Creating emphasis & suspense in your writing

**DOI:** 10.1007/s40037-019-00556-1

**Published:** 2019-12-18

**Authors:** Lorelei Lingard

**Affiliations:** Centre for Education Research & Innovation and Department of Medicine, Health Sciences Addition, Schulich School of Medicine & Dentistry-Western University, London, Canada

*Well-timed silence hath more eloquence than speech – Martin Tupper*

In the Writer’s Craft section we offer simple tips to improve your writing in one of three areas: Energy, Clarity and Persuasiveness. Each entry focuses on a key writing feature or strategy, illustrates how it commonly goes wrong, teaches the grammatical underpinnings necessary to understand it and offers suggestions to wield it effectively. We encourage readers to share comments on or suggestions for this section on Twitter, using the hashtag: #how’syourwriting?

Winston Churchill is said to have annotated his speeches with reminders to himself about rhythm and tempo—when to be silent, when to appear to struggle for the right word, when to pause for audience response (whether heckling or applause). His oratory, as a consequence, felt more like psalm than prose. Like other effective public speakers, Churchill knew that what is *not* said impacts the audience as much as what is. A pregnant pause whets appetites. An unanswered question hangs heavily. An abrupt redirection defers logical resolution. Wouldn’t it be lovely if writing could offer the same possibility?

It can. Prose need not always be a fast-flowing faucet, and readers need not be continuously engulfed. Pacing, pause and silence are important tools in your writing. Knowing the conventions of punctuation and syntax allows you to bend them strategically in order to both help your readers pay attention and enlist them into productive engagement with your ideas. This Writer’s Craft describes how to use punctuation and sentence structure to create emphasis and suspense in your research writing.

## Punctuation

Punctuation is not only grammatical; it is also rhetorical. You can use it for persuasion, to shape the reader’s experience. Because all forms of punctuation temper the forward motion of your prose, they are tools for pacing and silence, for producing anticipation and resolution, curiosity and satisfaction. Periods and exclamations are the strongest punctuation, bringing the reader to a complete stop, a sense of an idea resolved. While a question mark creates a complete stop, it has the opposite effect. It implies an answer, creating a moment of silence in the prose that produces suspense. You can extend that silence, perhaps by ending the paragraph with a question and answering it in the next paragraph. Or you can shorten the silence by providing an immediate answer:

Too often, our development as writers relies on trial and error, haphazard feedback, accidental improvements. Sad, but does it matter?

Too often, our development as writers relies on trial and error, haphazard feedback, accidental improvements. Sad, but does it matter? Only if we hope to find joy in our work.

Colons also allow you to instil anticipation: they’re like an intake of breath before revealing an illustration or a list. The ellipsis creates a drifting pause but … likely an unusual technique to find in a scholarly manuscript. Commas and semi-colons are a lighter harness but still curb the headlong flow of your prose. Commas, with their origins in antiquity and the elocutional school of punctuation [1], indicate intonation and pauses in oral speech, and they retain some of this flavour in writing. Semi-colons in particular help signal shifts in logic. Used properly, they tell the reader to gather herself for a development; however, used poorly, they send her on fruitless searches for mid-sentence meaning.

Work-based learning is central to postgraduate medical education; ethical issues rarely get attention.

In this sentence, the semi-colon presents a moment of intense work for the reader, who must ascertain what ethical issues have to do with the centrality of work-based learning in postgraduate medical education. The semi-colon’s promise of logical development remains unfulfilled and the reader is left wondering. *That* kind of pause you don’t want to cultivate.

Punctuating for pace is also a way to craft a particular writer’s voice in a piece. Seeking drama? Try short sentences. Their strong, staccato pacing results as much from the pauses between them as from their brevity. Trying for a conversational tone? Brackets take the reader on a brief detour from the main idea and create a sense of (hopefully purposeful) meandering. Similarly, the em dash—famously loved or hated—takes a momentary sidestep away from the main logic. Use it to set up whispered asides or provocative barbs. Take care though: brackets and dashes are boutique punctuation tools that can annoy the reader. Brackets, because they leave the reader to infer how the bracketed material integrates into the argument, can imply lazy thinking if you overuse them. Ask a trusted reader for honest feedback if you have tried to use punctuation for rhetorical effect and you wonder if you might have crossed the line.

## Syntax

Syntax is the set of rules, principles, and processes that govern how we construct sentences. Three aspects of syntax—sentence structure, subject-verb placement, and word order inversion—are described below as tools for pacing and emphasis.

First, you can employ sentence types to control the pacing of your prose. In a previous Writer’s Craft, we considered the structure and purposes of three sentence types: simple, compound and complex [2]. Consider this example:

My scholarly life is inextricably bound up with words. I write them to understand my research results. I read them to cultivate my ideas. I speak them to engage my colleagues. I publish them to share my knowledge.

Because all of these sentences are simple in their structure, there is a sense of strong, regular pacing. Parallelism enhances this pacing by creating a metronomic rhythm through the repeating subject/verb/object construction: ‘I write them’, ‘I read them’, ‘I speak them’, ‘I publish them’. Of course, you can’t continue ad nauseam in this pacing or your prose will sound like a primary school, Dick-and-Jane reader, plodding along rather than marching ahead. Pacing only works rhetorically when it is purposeful and varied. So perhaps we should quicken the pace in the next sentence by distilling the parallel structure down to two parallel lists of verbs:

My scholarly life is inextricably bound up with words. I write them to understand my research results. I read them to cultivate my ideas. I speak them to engage my colleagues. I publish them to share my knowledge. I love, covet and despise them; I create, destroy and mourn them.

It is not so much the simple sentence structure in this example that creates the pacing, it is the combination of simple structure, parallelism and distilling of the pattern.

Sentence structure doesn’t *dictate* pace; it’s a tool you use to *create* pace. Consider the following three sentences. They are all complex in structure, which means they have a main clause and a subordinate clause. The subordinate clause is just that—it is subordinate to, its information assigned less importance than that in the main clause. The subordinate clause is also transportable: you can put it at the beginning, the end, or somewhere mid-sentence.

Direct observation remains a rare commodity in medical education, although we insist on its importance for authentic feedback.

Although we insist on its importance for authentic feedback, direct observation remains a rare commodity in medical education.

Direct observation, although we insist on its importance for authentic feedback, remains a rare commodity in medical education.

The decision about how to position the two clauses should not be random. Rather, it should reflect the ‘given-new principle’ governing the ordering of information in a sentence, which states that known (given) information should precede new in order to maximize cohesion [3]. Each of the above examples, therefore, suggests a different assumption about what the reader already knows, and what is new to them. If we make such assumptions consciously as writers, we can play with emphasis and suspense.

Another method to create emphasis in your writing is by altering conventional subject/verb positioning. In English, the subject and the verb are the dominant meaning slots in the sentence, and the convention is to place them side by side in order to decrease the reader’s cognitive load. Every convention, however, presents a rhetorical opportunity to improvise for effect. Purposefully separating the subject and verb can create emphasis because the inserted phrase elaborates on the just-introduced subject, insisting the reader pause there rather than immediately landing on the verb.

Qualitative research methods, rare twenty years ago in medical education, have now become a popular approach to knowledge creation in our field.

Be careful not to create too much distance between subject and verb when you use this technique. As Sword cautions, when we separate subject and verb ‘by more than about a dozen words, readers quickly lose the plot’ (61) [4].

Another way to play with syntax for emphasis is word order inversion. Every language falls into one of six word-order types [5]. English belongs to the ***subject***-**verb**-*object* type, in which the natural order is to place the subject first. Alter that order, referred to as ‘inversion’, and you change the emphasis:

***We*value ***reliability and validity* above all else in high stakes assessments.

*Reliability and validity****we *value** above all else in high stakes assessments.

When you invert subject-verb-object word order, listen carefully for what happens to tone:

We need to change if our educational practices are going to embrace medicine’s humanity. And **change *we*will**.

This example moves part of the verb before the subject to emphasize the word ‘change’. But it perhaps sounds a bit … archaic? Yoda-esque? Knowing some of the more common uses of inversion can help you adopt this technique without just sounding odd. Inversion is used with negative adverbs:

Rarely **does*the medical education literature*** position itself socio-historically.

It may also be used when a sentence starts with an adverbial expression of place:

From the sciences **comes*our expectation of objectivity;*** from the humanities **comes*our embrace of subjectivity***.

Inversion is how we create questions:

**Are*entrustment decisions*** more reliable than conventional assessments?

And it is used with ‘so + adjective’ constructions:

So ubiquitous **is*the call for more direct observation***, that the act of questioning its feasibility in clinical training seems somewhat sacrilegious.

Because each of these inversion techniques works implicitly, through the *absence* of an expected pattern, they offer tools for creating subtle emphasis.

## Conclusion

Pacing, pause and silence are important tools in your writing. Knowing the conventions of punctuation and syntax allows you to bend them strategically in order to both help your readers pay attention and enlist them into productive engagement with your ideas. Remember though, there is a fine line between cultivating a curious, engaged reader and creating a frustrated, disengaged one. Pacing is a delicate aspect of the writer’s craft. Use it wisely.
